# Shape dependency of gold nanorods through TMB^2+^-mediated etching for the visual detection of NT-proBNP

**DOI:** 10.1039/d3ra00280b

**Published:** 2023-04-03

**Authors:** Chenlong Jiang, Xiangde Lai, Feng Han, Zhijun Gao, Haixia Yang, Xuan Zhao, Huajie Pang, Bin Qiao, Hua Pei, Qiang Wu

**Affiliations:** a Department of Clinical Laboratory of the Second Affiliated Hospital, School of Tropical Medicine, Key Laboratory of Emergency and Trauma of Ministry of Education, Research Unit of Island Emergency Medicine, Chinese Academy of Medical Sciences (No. 2019RU013), Hainan Medical University Haikou 571199 China hy0211085@hainmc.edu.cn phzmh61@aliyun.com wuqiang001001@aliyun.com; b Department of Clinical Laboratory of the First Affiliated Hospital, Hainan Medical University Haikou 570102 China

## Abstract

Heart failure (HF) is a major public health problem triggered by heart circulation disorders. Early detection and diagnosis are conducive to the prevention and treatment of HF. Hence, it is necessary to establish a simple and sensitive method to monitor the diagnostic biomarkers of HF. The N-terminal B-type natriuretic peptide precursor (NT-proBNP) is acknowledged as a sensitive biomarker. In this study, a visual detection method for NT-proBNP was developed based on the oxidized 3,3′,5,5′-tetramethylbenzidine (TMB^2+^)-mediated etching of gold nanorods (AuNRs) and double-antibody-sandwich ELISA. The etching color for different amounts of NT-proBNP was obvious and significant differences could be ascertained based on the blue-shift of the longitudinal localized surface plasmon resonance (LLSPR) of the AuNRs. The results could be observed by the naked eye. The constructed system showed a concentration range from 6 to 100 ng mL^−1^ and a low detection limit of 6 ng mL^−1^. This method exhibited negligible cross-reactivity toward other proteins, and the recoveries of the samples ranged from 79.99% to 88.99%. These results demonstrated that the established method is suitable for the simple and convenient detection of NT-proBNP.

## Abstract 2

Visual detection of NT-proBNP based on the principle of HRP-catalyzed TMB conversion into TMB^2+^ to mediate gold nanorods etching and an ELISA system.

## Graphical abstract

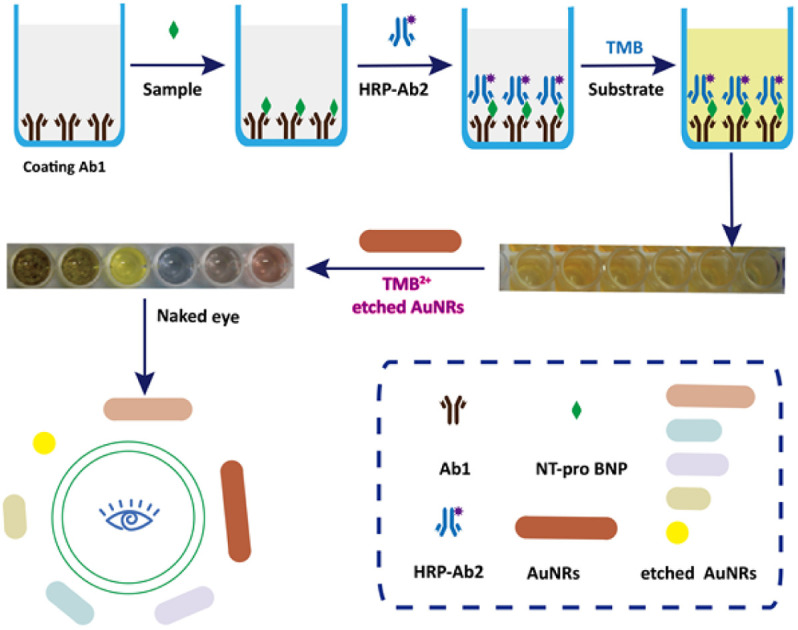

## Introduction

Heart failure (HF) is a condition that affects 26 million individuals globally and has recently emerged as the leading cause of cardiovascular disease-related mortality and morbidity.^1^ An aging population will result in a major rise in the prevalence of HF and associated healthcare costs. N-terminal pro-B-type natriuretic peptide (NT-proBNP) is currently regarded as the gold standard for the diagnosis and prognosis of HF.^2–5^ Therefore, it is crucial to create a sensitive, specific, and precise NT-proBNP detection technique.

Traditional immunoassays as well as contemporary biosensors are two of the analysis techniques that have been developed and used to detect NT-proBNP. The usage of earlier techniques, such as enzyme-linked immunosorbent assay (ELISA) and immunochromatographic test, is constrained by the need for basic equipment, like microplate readers and strip readers.^6–12^ The latter assays include electrochemical biosensors, photoelectrochemical biosensors, surface plasma resonance biosensors, electrochemiluminescence biosensors, and microfluidic immunoassay biosensors, among others.^13–22^ Large instruments are necessary for these delicate biosensors, which are expensive, uncomfortable for users, and need trained technicians. Due to these flaws, it is a challenge to meet the demand for NT-proBNP monitoring in insufficient experimental settings and less developed countries.

Naked-eye colorimetric detection based on nanomaterials has recently drawn a lot of interest. For instance, assays of Ochratoxin A, *E. coli* O157, PSA, and HIV-1 capsid antigen p24 have been studied.^23–25^ Gold nanoparticles (AuNPs) are good signal indicator, which can easily cause dramatic color change by slightly changing their shapes, sizes, compositions and aggregation states. The AuNPs solution obtained with an analyte target was found to produce a single color shift that was plainly visible in the reaction solution. As a result, it might be challenging to discriminate between fluctuations in optical density (without a spectral peak shift) and to precisely measure the concentration of the target molecules by the naked eye. The ability of human eyes to detect subtle color differences is well recognized (with spectral peak shifts). Gold nanorods (AuNRs) have been used as a colorful chromogenic substrate to detect a large number of target molecules because AuNRs display different multicolored and variable longitudinal plasmon wavelengths.^26^

The morphology of AuNRs obtained through oxidized 3,3′,5,5′-tetramethylbenzidine (TMB^2+^) generated by HRP and the idea of antigen–antibody combination were used in this demonstration to showcase a portable and visible detection method for quantifying NT-proBNP.

## Experimental

### Materials and reagents

NT-proBNP antigen, coating antibody for NT-proBNP (Ab1), and detection antibody for NT-proBNP (Ab2) were supplied by Nanjing Oukai Biotechnology Co., Ltd (Nanjing, China). ELISA plates were obtained from Sangon Biotech (Shanghai, China) Co., Ltd. (Shanghai, China). Bovine serum albumin (BSA) was supplied by Beijing Solarbio Science & Technology Co., Ltd. (Beijing, China). Ultrapure water (≥18.2 MΩ) was produced by a Millipore water purification system and used in all the solutions. 10 mmol L^−1^, pH 7.4 phosphate-buffered saline (PBS) containing 0.05% tween-20 (PBST) was obtained from Labgic Technology Co., Ltd. (Beijing, China). The activated HRP reagents kit was obtained from Jinan Xingbao Biotechnology Co., Ltd. (Jinan, China). 3,3′,5,5′-Tetramethylbenzidine (TMB) and other chemical reagents were of analytical grade and purchased from Aladdin (Shanghai, China). The negative serum came from the Second Affiliated Hospital of Hainan Medical University, which was approved by the ethics committee (HYLL-2022-419).

### Instruments

The images of the surface morphology of the gold nanorods (AuNRs) were obtained using an Hitachi HT 7800 transmission electron microscopy system (TEM) (Hitachi, Japan). An incubator was purchased from Shanghai Bosun Industrial Co., Ltd. The constant temperature water bath was provided by Shanghai Yiheng Scientific Instrument Co., Ltd. The blue-shifts of the longitudinal localized surface plasmon resonance (LLSPR) of the AuNRs were measured using a Bio-Tek Synergy HTX microplate reader (Winooski, USA), and using a Vortex Mixer purchased from Eppendorf Company (Hamburg, Germany).

### Synthesis of AuNRs

AuNRs were prepared according to the literature with several modifications.^27^ Briefly, (1) the seed liquid was prepared as follows: 2.5 mL of 0.2 M cetyltrimethylammonium bromide (CTAB) was transferred into a 10 mL conical flask and frequently stirred at 400 rpm min^−1^ with a magnetic stirrer. Then, 2.5 mL of 0.5 mM HAuCl_4_ solution was added. Subsequently, 0.3 mL of 0.01 M freshly prepared NaBH_4_ solution was added. The mixed liquid was incubated at 30 °C for 30 min; (2) the growth solution was prepared as follows: 9 g of CTAB and 250 mL of ultrapure water were added into a conical flask, with continuous heating and stirring until the CTAB was completely dissolved, and afterward, 1.1 g of 5-bromosalicylic acid was added. After stirring for 5 min, 12 mL of 4 mM AgNO_3_ solution was added to the solution and incubated at 30 °C for 15 min. Then, 250 mL of 1 mM HAuCl_4_ solution was added, and the solution gradually turned orange-red. Subsequently, 2 mL of 0.064 M ascorbic acid (AA) was dropped into the conical flask. The color of the mixed solution rapidly changed from orange-red to colorless. Afterward, 800 μL of seed liquid was added and mixed for 30 s. Finally, the conical flask was left undisturbed at 30 °C for 24 h. After that, the solution was centrifuged at 8000 g min^−1^ for 15 min, and the supernatant was removed. The AuNRs precipitate was resuspended with purified water and placed in a 30 °C incubator for further use.

### HRP-labeled Ab2 (HRP-Ab2)

The HRP was labeled with Ab2 according to the kit instructions for activating HRP reagents. Briefly, 25 μL of Ab2 (4 mg mL^−1^) was transferred into a 1.5 mL tube. Then, 100 μL reagent I activated HRP (10 mg mL^−1^) was added. The pH of the mixture solution was adjusted to 9.5 with reagent II (about 20 μL) and then reacted for 1 h at 37 °C. Afterward, reagent III was dropped into the tube and stored at 4 °C for further use.

### Procedures for the visual detection of NT-proBNP

Ab1 in pH 9.6 carbonate bicarbonate buffer was added into 96-well plates for incubation at 37 °C. Then, each well was washed three times with pH 7.2 PBS containing 0.05% tween 20 (PBST). Subsequently, different concentrations of NT-proBNP (in PBS buffer containing 5% BSA) were added to the plates and incubated at 37 °C. After three times washing, each well was dispensed with 100 μL HRP-Ab2 and incubated at 37 °C. After further washing, TMB substrate solution was pipetted into each well and reacted at 37 °C. Then, the reaction was stopped by adding 2 M H_2_SO_4_. After that, AuNRs solution was placed into each well and reacted at 37 °C. Finally, photographs were taken by a smartphone and the corresponding absorbance at 500–900 nm was measured using a Bio-Tek Synergy HTX microplate reader.

### Optimization of the experimental conditions

To obtain high sensitivity, several experimental parameters of the proposed NT-proBNP assay were optimized by altering the following: coating time from 30 min to 120 min; incubation time for NT-proBNP from 30 min to 120 min; incubation time for HRP-Ab2 from 30 min to 120 min; and etching time for AuNRs from 5 min to 20 min.

### Establishment of the NT-proBNP standard colorimetric card

The standard multicolor card was established by adding different concentrations of NT-proBNP according to the procedures for the visual detection of NT-proBNP under the above optimization conditions, except the concentration of NT-proBNP added ranged from 3 to 100 ng mL^−1^. Meanwhile, the LLSPR peak shift of the AuNRs was recorded using a Bio-Tek Synergy HTX microplate reader.

### Method specificity

To demonstrate the specificity of the purposed method, serum amyloid A antigen (SAA), C-reactive protein (CRP), heparin-binding protein antigen (HBP), P1 protein of mycoplasma pneumoniae (P1), and P30 protein of mycoplasma pneumoniae (P30) with a concentration of 20 ng mL^−1^ were tested. The operation steps were the same as in the detection procedure for the establishment of the NT-proBNP standard colorimetric card.

### Detection of NT-proBNP in the serum samples

To verify the accuracy and feasibility of the established method for serum samples, three different concentrations of NT-proBNP were spiked in 10-fold diluted healthy human serum without NT-proBNP. The experimental steps were the same as those for the establishment of the NT-proBNP standard colorimetric card.

## Results and discussion

### Characterization of AuNRs

**Fig. 1:**
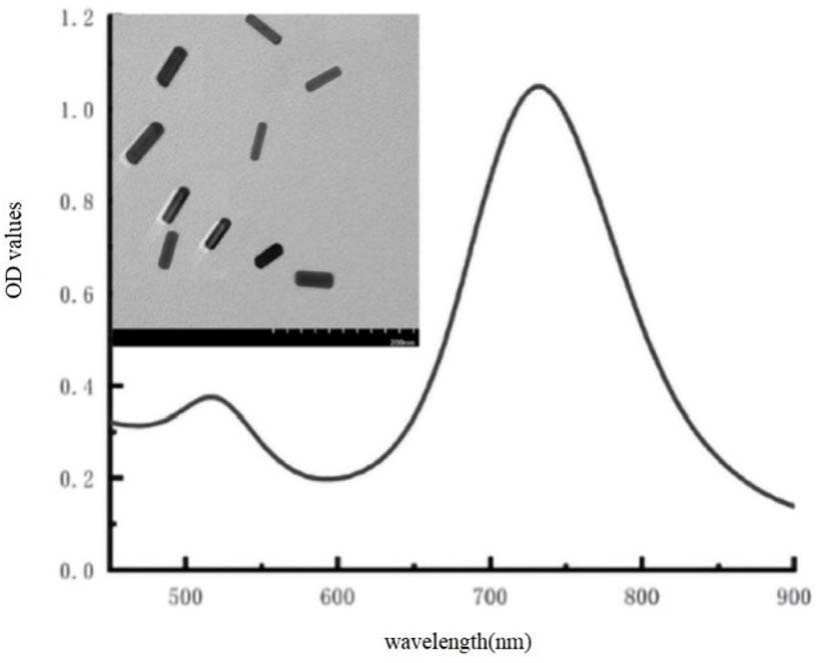
UV-vis absorption spectra and TEM image of the original AuNRs.

As shown in Fig. 1, AuNRs were created using a seed-mediated approach and were identified by their TEM images and UV-vis absorption. The TEM picture of the produced AuNRs revealed good consistency. The typical longitudinal and transverse bands were found in the UV-vis absorption spectrum at wavelengths of 520 nm and 730 nm, respectively. These findings show that the AuNRs were properly prepared.

### Principle behind the established visual detection of NT-proBNP

**Fig. 2:**
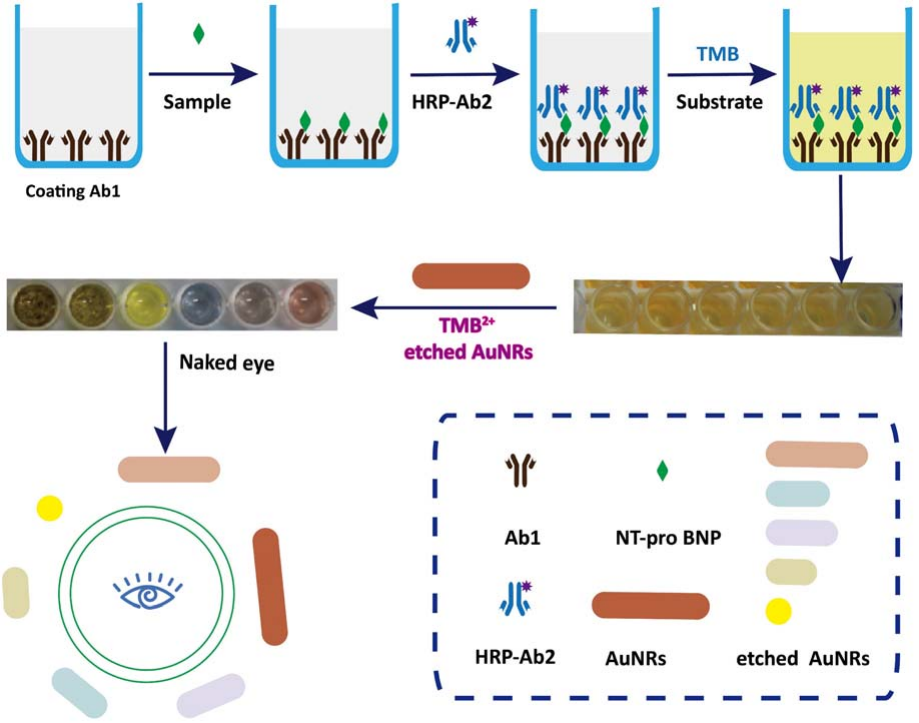
The principle of the visual detection of NT-proBNP.

**Fig. 3:**
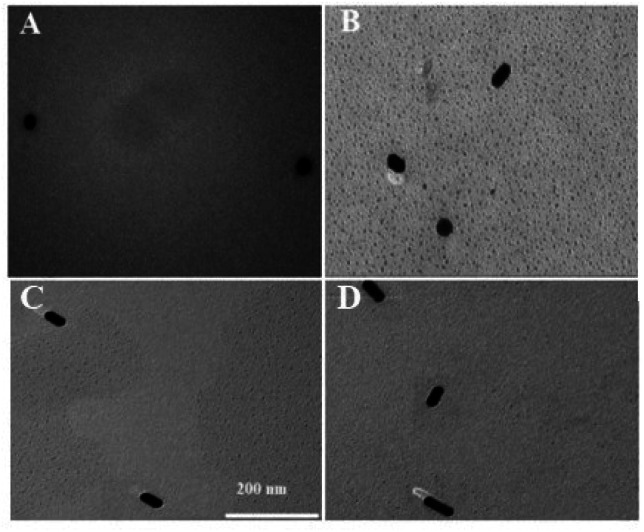
TEM image of AuNRs in the presence of (A) 100 ng mL^−1^ (B) 50 ng mL^−1^ (C) 25 ng L mL^−1^, and (D) 12.5 ng mL^−1^ NT-proBNP.

The principle of visually detecting NT-proBNP was illustrated in Fig. 2. A sandwich ELISA assay was employed in our investigation. HRP-Ab2 was immobilized on the wells after the formation of the double-antibody-sandwich immune complex. The amount of HRP immobilized on the wells was positively proportional to the concentration of NT-proBNP in the sample. Then, TMB^2+^ (oxidized TMB) was produced by TMB in the presence of HRP. Finally, AuNRs solution was added in and shortened by the etching effect of TMB^2+^ in the presence of CTAB, which caused a blue-shift of the longitudinal localized surface plasmon resonance (LLSPR) peak, while the transverse localized surface plasmon resonance (TLSPR) peaks were nearly unaffected. Fig. 1 shows the TEM images and UV-vis spectra of the prepared AuNRs. The LSPR and TSPR of the AuNRs were located at 730 nm and 520 nm, respectively. The length of the AuNRs was about 60 nm. With the addition of various concentrations of NT-proBNP, the length of the AuNRs was shortened by various degrees. The TEM images are shown in Fig. 3. These results demonstrate the proposed method is feasible and can be used for the quantitative detection of NT-proBNP.

### Optimization of the conditions of the immunosensor

**Fig. 4:**
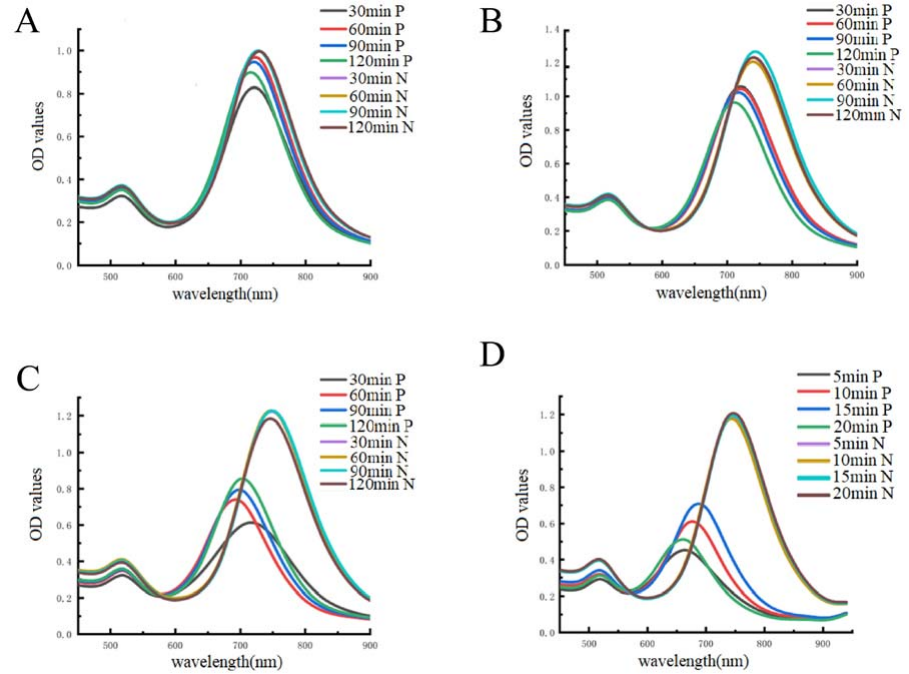
Effect of the incubation time of (A) Ab1 (B) NT-proBNP (C) HRP-Ab2, and (D) AuNRs on the proposed assay of NT-proBNP. P indicates the sample containing NT-proBNP; N indicates the sample without NT-pro BNP.

The incubation times of Ab1, NT-proBNP, and HRP-Ab2 were all tuned in order to achieve the best sensitivity. The optimizated results are shown in Fig. 3A–D. The incubation times for Ab1, NT-proBNP, HRP-Ab2, and AuNRs were 120, 120, 60, and 20 min, respectively, according to the creation of a striking blue-shift (Fig. 4).

### Calibration curve for NT-proBNP detection

**Fig. 5:**
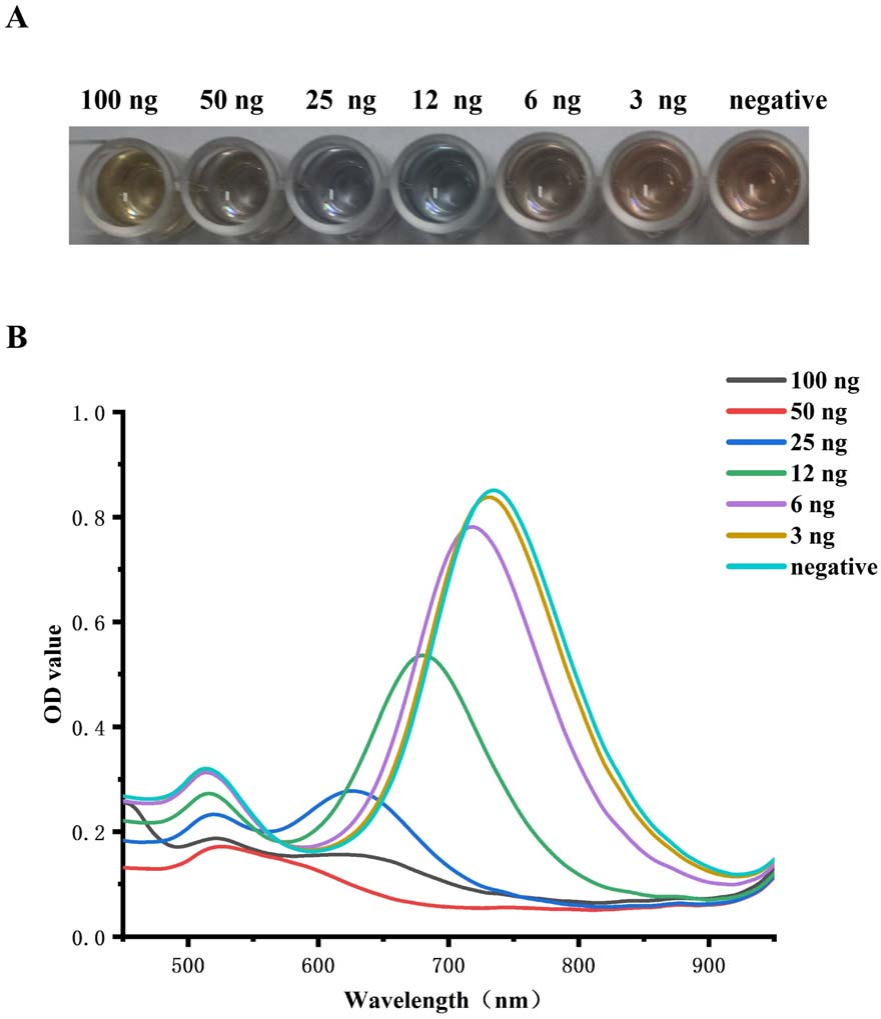
(A). Standard color card for the semi-quantitative determination of NT-pro BNP. (B). LLSPR extinction peaks shift with the different NT-proBNP concentrations.

With the rise in NT-proBNP concentration from 6 to 100 ng mL^−1^, an extraordinary color change from brown to blue and ultimately to yellow was clearly visible to the unaided eye, as shown in Fig. 5A. The color of the well of 3 ng mL^−1^ was just like the color of the negative well, which was brown. Meanwhile, the LLSPR peak gradually shifted from 730 nm to 520 nm with the increase in NT-proBNP concentration, as shown in Fig. 5B. The calibration plots show the good linear relationship between the blue-shifts of the LLSPR peak (Δ*λ*_max_) and NT-proBNP concentration in the range from 6 to 50 ng mL^−1^, *y* = 4.1036*x* − 4.3271 (*R*^2^ = 0.99). Hence, the visual assay established could be applied to evaluate NT-proBNP concentrations in deficient experimental conditions.

### Specificity of the established immunosensor

**Fig. 6:**
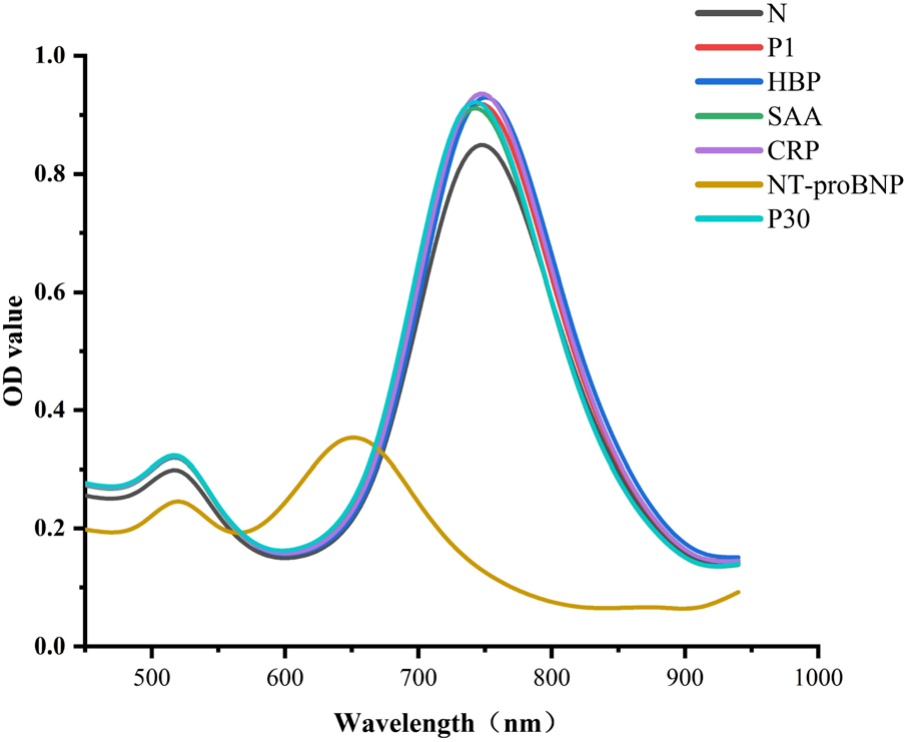
Specificity for the visual detection of NT-pro BNP assay against 20 ng mL^−1^ of NT-pro BNP, SAA, CRP, HBP, P1, and P30.

Several analogs were found using targeted investigations to gauge the immunosensor's specificity. In the presence of 20 ng mL^−1^ of NT-proBNP, SAA, CRP, HBP, P1, and P30, there was a peak shift of the LLSPR, as shown in Fig. 6. While the other proteins exhibited negligible peak-shifts, only 20 ng mL^−1^ of NT-proBNP clearly blue-shifted the LSPR of AuNRs. Because of this, the suggested immunosensor showed high selectivity for NT-proBNP.

### Recovery test of NT-proBNP in serum samples

To assess the effectiveness of the analytical process serum samples were spiked to 12, 25, and 50 ng mL^−1^. The findings in Table 1 show that the NT-proBNP recoveries fell between the ranges of 79.99% and 88.99%, indicating that the established optical detection for NT-proBNP had a high degree of accuracy.

**Table 1:** Recoveries of NT-proBNP from spiked serum samples by the proposed assay (*n* = 3)

Spiked concentration (ng mL^−1^)	Detected (ng mL^−1^)	Recovery (%)
50	44.49	88.99
25	21.07	84.29
12	9.59	79.99

## Conclusions

The conventional ELISA for detecting NT-proBNP, which has strong repeatability and specificity, was proposed to be more convenient in this study using a novel chromatic method based on AuNRs. The assay could be successfully recovered after being used to spike clinical samples. Unlike the conventional ELISA, this immunoassay can be applied in the absence of ideal experimental circumstances and can be visually identified without the use of specific equipment. Furthermore, by simply changing the relevant antigen and antibody, this approach can be quickly applied to other diagnoses.

## Conflicts of interest

The authors declare that they have no competing interests.

## Supplementary Material
